# Supplementary material for: A multi-model architecture based on deep learning for aircraft load prediction
Source: Commun Eng. 2023 Jul 18;2:47. doi: 10.1038/s44172-023-00100-4 (PMC10956017; doi:10.1038/s44172-023-00100-4)
Supplement: Supplementary file 2 — Supplementary Information [file 44172_2023_100_MOESM2_ESM.pdf]

Supplementary Information

A Multi-model Architecture Based on Deep Learning for Aircraft Load Prediction

Chenxi Sun[1,2], Hongyan Li[1,2*], Hongna Dui[3], Shenda Hong[4,5*], Yongyue Sun[1,2], Moxian Song[1,2], Derun Cai[1,2], Baofeng Zhang[1,2], Qiang Wang[3], Yongjun Wang[3] and Bo Liu[1,2]

[1]School of Intelligence Science and Technology, Peking University, Beijing, China.
[2]Key Laboratory of Machine Perception (Ministry of Education), Peking University, Beijing, China.
[3]The Aviation Industry Corporation of China, Ltd., Chengdu Aircraft Design&Research Institute, Chengdu, China.
[4]National Institute of Health Data Science, Peking University, Beijing, China.
[5]Institute of Medical Technology, Health Science Center of Peking University, Beijing, China.

*Corresponding author(s). E-mail(s): leehy@pku.edu.cn; hongshenda@pku.edu.cn;
Contributing authors: sun_chenxi@pku.edu.cn; leehy@pku.edu.cn; redhated@pku.edu.cn; songmoxian@pku.edu.cn; cdr@stu.pku.edu.cn; boffinzhang@stu.pku.edu.cn; 287640103@qq.com; 9561141@qq.com; liubo2022@stu.pku.edu.cn;

S1 Supplementary Methods

S1.1 Overall Process

The overall process of our method is shown in Figure S1.

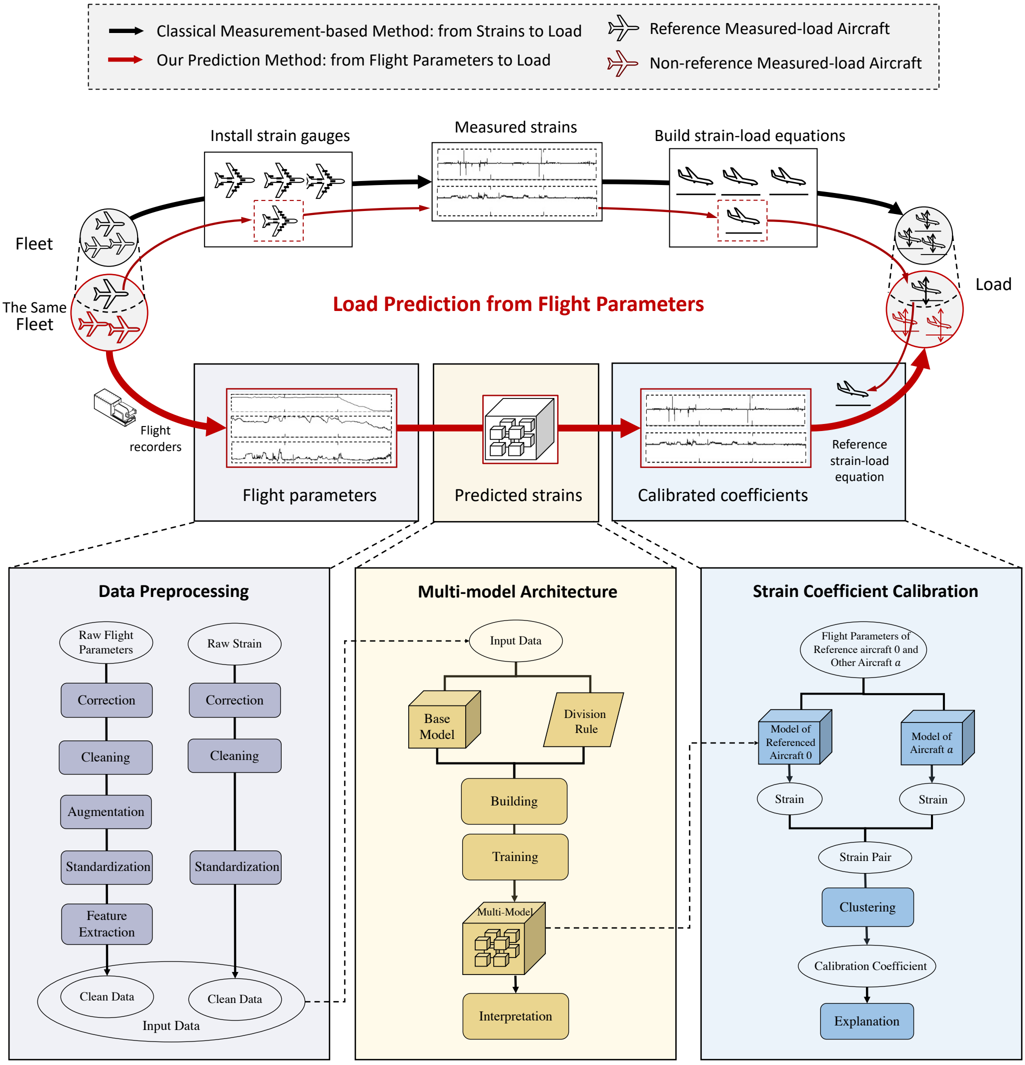

Fig. S1 Aircraft Load Prediction from Flight Parameters

Table S1 Notations and Description

Notation	Description
X, x_i, X^a	Flight parameters, a flight parameter feature, flight parameter of aircraft a
E, E_i, E^a	Strains, a strain, strain of aircraft a
F, F^a	Load, load of aircraft a
SF, SF_i^a	Calibration coefficient, SF of E_i between aircraft a and reference aircraft 0
f	Prediction model
θ, β, W, b	Model parameters (parameters, coefficients, weights, bias)
$\mathcal{L}, L$	Loss function
$\mathcal{E}$	Error between prediction and ground truth
$\mathcal{H}, \mathcal{I}, IV$	Entropy, mutual information, information entropy
S	Silhouette coefficient
R^2	Coefficient of determination

Table S2 Statics of Data and Features

	Features
Flight parameters (model input / independent variable)	Weight, Mach, Height(Altitude), Attack angle, Sideslip angle, Dynamic pressure, Normal overload(Nz), Lateral overload(Nx), Axial overload(Ny), Pitch angle, Roll angle, Heading angle, Roll angular velocity, Pitch angular velocity, Yaw angular velocity, Roll angular acceleration, Pitch angular acceleration, Yaw angular acceleration, Left canard wing deflection, Right canard wing deflection, Left flap deflection, Right flap deflection, Left outer aileron deflection, Right outer aileron deflection, Left inner aileron deflection, Right inner aileron deflection, Left rudder deflection, Right rudder deflection
Strains (model output / dependent variable)	Wing shear bridge-1, Wing shear bridge-2, Wing bending moment bridge-1, Wing bending moment bridge-3, Wing bending moment bridge-6, Canard shear bridge, Canard bending moment bridge-1, Vertical tail shear bridge, Vertical tail bending moment bridge-1, Fuselage bending moment bridge-1

Table S3 Statics of Records

Aircraft / Number	P123	P124	P125	P126	P127	Total
Flight test	20	21	20	20	19	100
Record	396,725	436,308	408,732	430,982	330,412	2,003,159

S1.2 Data Description

The dataset contains 2,003,159 records from 5 aircrafts, about 400,000 records per aircraft. Each record consists of 28 flight parameters and 10 strains. The statics of data and records is shown in Table S3. In one flight, the flight parameters and strains

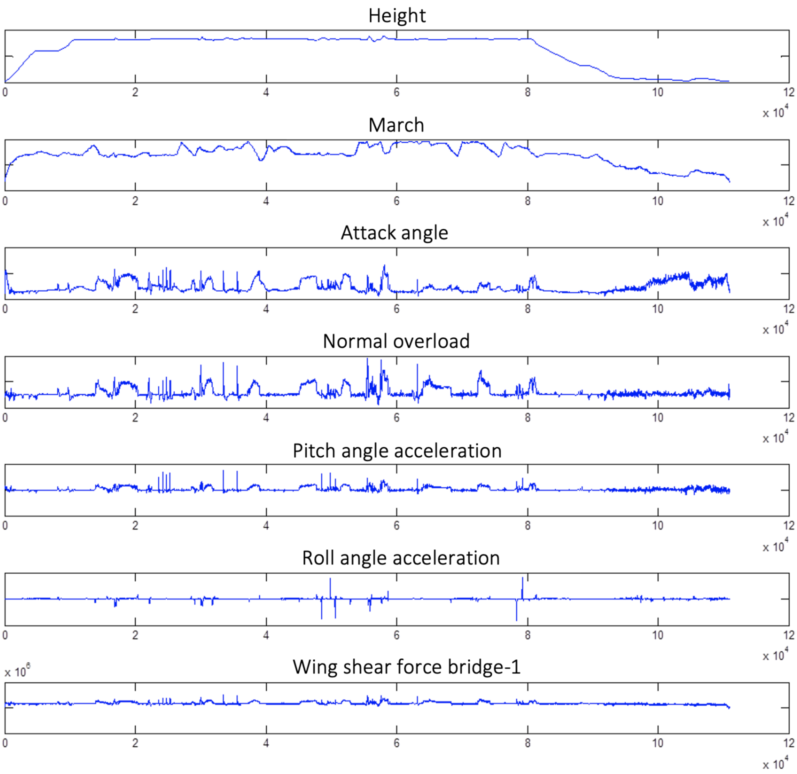

Fig. S2 Examples of 6 Flight Parameters and 1 Strain during One Flight

will form time series, as shown in Figure S2. At every sampling point, we use the flight parameter (model input / independent variable) to predict the strain (model output / dependent variable), as shown in Table S2.

S1.3 Granger Causality

S1.3.1 Classical Granger Causality Test

Finding the causal relationship between Flight parameters and strain is to determine the feasibility of using flight parameters to predict strains.

Flight parameters and strain data are in sequential format. Granger causality quantifies the past of one time series and helps to predict the future evolution of another time series. It defines causality from the perspective of prediction and can determine whether one time-series/sequence is the cause of another. We test the Granger Causality for our data: If the P value of F test in Granger causality tests from a flight parameter X to a strain E is less than 5%, we will conclude that there is the classical Granger causality from X to E. As shown in Table S6, more than More than 70% (227/308) pairs have Granger causality.

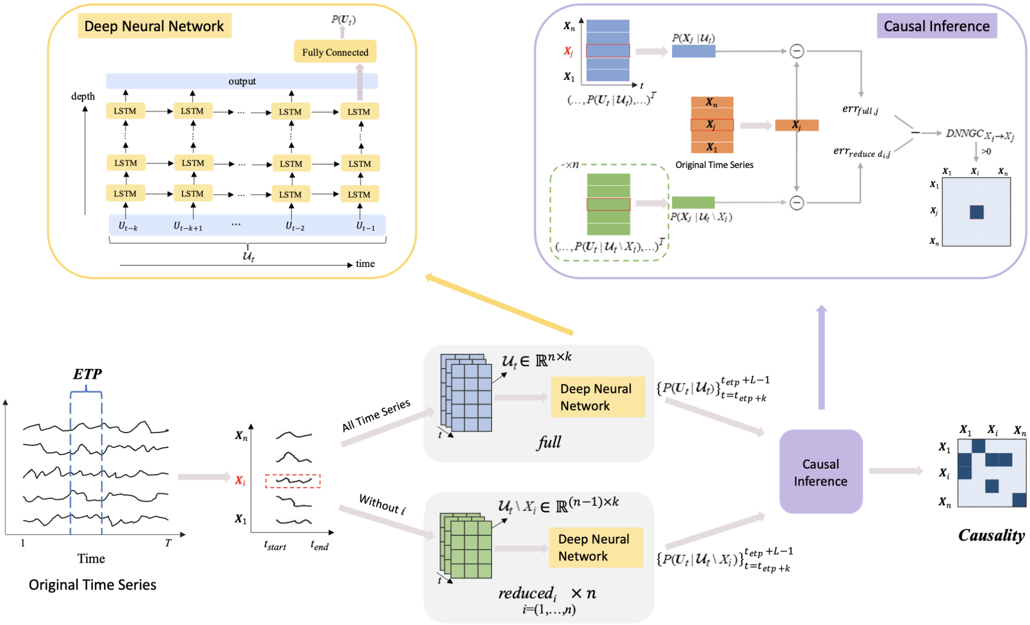

Fig. S3 Framework of Dynamic Granger Causality Analysis Method Based on Deep Learning

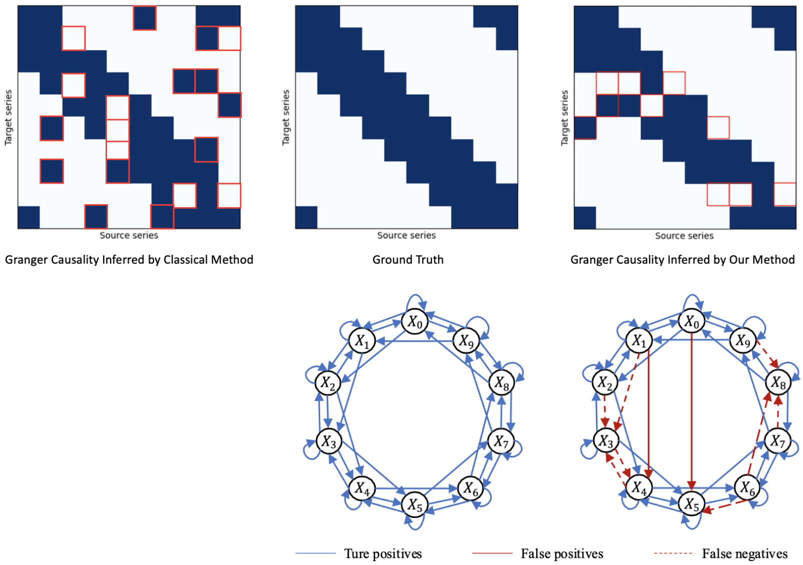

Fig. S4 Experimental Results of the Cyclic Granger Causality

Table S4 Statics of P Value of F Test in Granger Causality Tests
1-28 represent flight parameters; I-XI represent strains. Their names are in Table S2. The Granger causality tests are from flight parameters to strains. Boldface indicates that the p value is less than 5%. More than 70% (227/308) pairs have Granger causality.

	I	II	III	IV	V	VI	VII	VIII	IX	X	XI
1	0.54	0.418	**0.0**	0.32	0.482	0.722	**0.039**	0.932	0.556	0.423	**0.0**
2	**0.0**	**0.0**	0.531	**0.0**	**0.0**	**0.0**	**0.0**	**0.0**	**0.029**	**0.0**	0.18
3	**0.003**	**0.015**	**0.0**	**0.001**	**0.011**	**0.049**	**0.0**	**0.043**	0.875	0.104	0.06
4	0.057	**0.0**	0.113	0.179	**0.005**	**0.013**	**0.014**	0.421	**0.0**	**0.0**	**0.026**
5	0.82	**0.002**	**0.0**	0.798	0.066	**0.0**	0.523	0.786	**0.0**	**0.0**	**0.001**
6	**0.0**	**0.0**	0.303	**0.0**	**0.0**	**0.0**	**0.0**	**0.0**	**0.007**	**0.0**	0.477
7	**0.0**	**0.0**	0.056	**0.0**	**0.0**	**0.0**	**0.0**	**0.0**	**0.0**	**0.0**	0.742
8	**0.001**	**0.0**	**0.0**	**0.0**	**0.0**	**0.0**	0.306	**0.022**	**0.0**	**0.0**	0.546
9	0.738	**0.003**	**0.001**	0.829	0.236	0.298	0.306	0.635	**0.0**	**0.0**	**0.0**
10	0.129	**0.002**	**0.0**	0.081	**0.02**	**0.01**	**0.037**	**0.014**	**0.0**	**0.0**	0.799
11	0.877	0.601	0.063	0.74	0.676	0.171	0.646	0.296	**0.0**	**0.0**	**0.028**
12	0.103	**0.047**	**0.037**	0.203	0.098	0.423	0.17	0.212	0.242	0.05	**0.0**
13	0.094	**0.0**	**0.003**	**0.02**	**0.0**	**0.0**	0.092	**0.015**	**0.0**	**0.0**	0.21
14	**0.0**	**0.0**	0.467	**0.0**	**0.0**	**0.0**	**0.0**	**0.0**	**0.0**	**0.0**	0.436
15	0.46	**0.0**	0.142	0.29	**0.003**	**0.0**	0.391	0.86	**0.0**	**0.0**	**0.013**
16	**0.0**	**0.0**	**0.0**	**0.0**	**0.0**	**0.0**	**0.0**	**0.0**	**0.0**	**0.0**	**0.008**
17	**0.0**	**0.0**	**0.0**	**0.0**	**0.0**	**0.0**	**0.0**	**0.0**	**0.0**	**0.0**	**0.0**
18	**0.0**	**0.0**	**0.0**	**0.0**	**0.0**	**0.0**	**0.004**	**0.001**	**0.0**	**0.0**	**0.038**
19	**0.0**	**0.0**	0.841	**0.0**	**0.0**	**0.0**	**0.0**	**0.0**	0.677	0.108	**0.0**
20	**0.0**	**0.0**	0.835	**0.0**	**0.0**	**0.0**	**0.0**	**0.0**	0.687	0.11	**0.0**
21	**0.002**	**0.0**	**0.047**	**0.018**	**0.0**	**0.0**	**0.003**	0.117	**0.0**	**0.0**	0.057
22	**0.002**	**0.0**	**0.048**	**0.02**	**0.0**	**0.0**	**0.003**	0.123	**0.0**	**0.0**	0.058
23	**0.0**	**0.0**	**0.0**	**0.0**	**0.0**	**0.0**	0.737	0.266	**0.0**	**0.0**	**0.0**
24	**0.0**	**0.0**	**0.0**	**0.0**	**0.0**	**0.001**	**0.001**	**0.011**	**0.0**	**0.0**	**0.0**
25	**0.0**	**0.0**	**0.0**	**0.0**	**0.0**	**0.0**	0.704	0.278	**0.0**	**0.0**	**0.0**
26	**0.0**	**0.0**	**0.0**	**0.0**	**0.0**	**0.0**	**0.001**	**0.011**	**0.0**	**0.0**	**0.0**
27	**0.0**	**0.0**	**0.0**	**0.0**	**0.0**	**0.0**	**0.0**	**0.002**	**0.0**	**0.0**	**0.012**
28	0.84	**0.0**	**0.0**	0.202	**0.003**	**0.0**	0.07	0.88	**0.0**	**0.0**	**0.017**

Table S5 The Accuracy of Strain Forecasting Using Flight Parameters Inferred by the Classical Granger Causality Method and the Deep Learning-based Granger Causality Method

Aircraft / Data	P123	P124	P125	P126	P127
Classical Granger Causality	74.53%	80.01%	82.99%	80.14%	85.45%
Deep Learning-based Granger Causality	**83.23%**	**84.97%**	**83.75%**	**85.00%**	**86.34%**

S1.3.2 Deep Learning-based Granger Causality Test

Flight parameters are multivariate time series from complex system, which makes the test difficult to implement: (1) The classical Granger causality only analyzes two variables and ignores the influence among multiple variables; (2) The prior knowledge assumes that the relation between variables is linear and can not analyze the complex nonlinear dependency in system; (3) The method only analyzes static causality but omits the potential dynamic causality.

Table S6 Statics of Error Improvement in Granger Causality with LSTM model.
1-28 represents flight parameters; I-XI represents strains. Their names are in Table S2. The Granger causality tests are from flight parameters to strains. Boldface indicates there is improvement in MES Error when predicting the strain after adding the flight parameter as model input. More than 80% (259/308) pairs have deep learning-based Granger causality.

	I	II	III	IV	V	VI	VII	VIII	IX	X	XI
1	0.016	**-0.011**	**-0.005**	**-0.039**	**-0.048**	**-0.019**	**-0.005**	0.037	**-0.009**	0.049	**-0.003**
2	**-0.036**	0.0	0.02	**-0.02**	**-0.016**	**-0.007**	**-0.029**	**-0.001**	**-0.037**	**-0.014**	0.004
3	**-0.048**	**-0.011**	**-0.05**	**-0.008**	**-0.004**	**-0.038**	**-0.047**	**-0.003**	**-0.045**	**-0.022**	**-0.044**
4	**-0.008**	**-0.009**	**-0.046**	**-0.042**	**-0.017**	**-0.032**	**-0.025**	**-0.043**	0.0	**-0.002**	**-0.049**
5	0.007	**-0.045**	**-0.019**	0.035	**-0.043**	**-0.037**	**-0.014**	**-0.043**	**-0.009**	**-0.01**	**-0.019**
6	**-0.005**	**-0.006**	**-0.032**	**-0.031**	**-0.013**	**-0.006**	**-0.023**	**-0.008**	**-0.032**	**-0.032**	0.042
7	**-0.007**	**-0.033**	0.042	**-0.049**	**-0.024**	**-0.022**	**-0.035**	**-0.013**	**-0.022**	**-0.04**	0.013
8	**-0.004**	**-0.028**	**-0.04**	**-0.043**	**-0.021**	**-0.012**	0.018	**-0.007**	**-0.0**	**-0.021**	**-0.039**
9	0.033	**-0.004**	**-0.025**	**-0.036**	0.006	0.04	0.038	0.019	**-0.02**	**-0.045**	**-0.02**
10	0.028	**-0.001**	**-0.008**	**-0.015**	**-0.003**	**-0.022**	**-0.04**	**-0.047**	**-0.042**	**-0.002**	0.018
11	**-0.006**	0.005	0.009	0.023	0.037	**-0.045**	0.043	0.041	**-0.035**	**-0.011**	**-0.022**
12	0.025	**-0.027**	**-0.045**	**-0.026**	0.034	0.023	0.014	0.026	**-0.029**	**-0.017**	**-0.01**
13	**-0.046**	**-0.002**	**-0.038**	**-0.012**	**-0.042**	**-0.046**	**-0.05**	**-0.038**	**-0.026**	**-0.013**	0.011
14	**-0.027**	**-0.038**	0.026	**-0.031**	**-0.011**	**-0.028**	**-0.028**	**-0.024**	**-0.022**	**-0.038**	0.006
15	0.013	**-0.043**	**-0.047**	**-0.037**	**-0.043**	**-0.042**	0.046	0.028	**-0.004**	**-0.02**	**-0.001**
16	**-0.047**	**-0.014**	**-0.043**	**-0.023**	**-0.006**	**-0.012**	**-0.041**	**-0.012**	**-0.002**	**-0.047**	**-0.005**
17	**-0.048**	**-0.022**	**-0.003**	**-0.01**	**-0.029**	**-0.02**	**-0.03**	**-0.046**	**-0.012**	**-0.013**	**-0.043**
18	**-0.032**	**-0.006**	**-0.046**	**-0.041**	**-0.046**	**-0.027**	**-0.016**	**-0.022**	0.0	**-0.009**	**-0.011**
19	**-0.005**	**-0.002**	**-0.034**	**-0.019**	**-0.039**	**-0.041**	**-0.033**	**-0.022**	**-0.044**	**-0.032**	**-0.023**
20	**-0.001**	**-0.016**	0.011	**-0.037**	**-0.046**	**-0.026**	**-0.014**	**-0.034**	0.037	**-0.017**	**-0.033**
21	**-0.034**	**-0.018**	**-0.031**	**-0.035**	**-0.017**	**-0.002**	**-0.022**	**-0.045**	**-0.018**	**-0.0**	**-0.017**
22	**-0.032**	**-0.018**	**-0.014**	**-0.022**	**-0.003**	**-0.029**	**-0.009**	0.049	**-0.017**	**-0.032**	**-0.031**
23	**-0.0**	**-0.025**	**-0.015**	**-0.014**	**-0.039**	**-0.019**	0.006	0.033	**-0.041**	**-0.045**	**-0.027**
24	**-0.002**	**-0.018**	**-0.002**	**-0.033**	**-0.047**	**-0.041**	**-0.017**	**-0.035**	**-0.027**	**-0.006**	**-0.008**
25	**-0.008**	**-0.01**	**-0.02**	**-0.027**	**-0.009**	**-0.03**	**-0.033**	0.018	**-0.031**	**-0.045**	**-0.038**
26	**-0.047**	**-0.047**	**-0.02**	**-0.049**	**-0.016**	**-0.0**	**-0.002**	**-0.014**	**-0.023**	0.001	**-0.016**
27	**-0.033**	**-0.003**	**-0.045**	**-0.013**	**-0.012**	**-0.007**	**-0.008**	**-0.03**	**-0.013**	**-0.047**	**-0.001**
28	0.046	**-0.021**	**-0.005**	0.014	**-0.01**	**-0.01**	0.012	0.038	**-0.008**	**-0.006**	**-0.01**

Thus, to solve these issues, the deep learning-based Granger causality is proposed in our previous work as shown in Figure S3, which measures causality between two variables through a deep learning model: If the difference between the error of forecasting strain E with strain E as model input and that with both only strain E and flight parameter X as model input is bigger than 0 in Equation 1, we will conclude that there is the deep learning-based Granger causality from X to E. The model is Long Short-Term Memory (LSTM) [1].

$$\Delta_{\mathcal{E}} = \mathcal{E}(LSTM(E, X), \hat{E}) - \mathcal{E}(LSTM(E), \hat{E}) < 0 \quad (1)$$

The prediction model is Long Short-Term Memory (LSTM) [1]. LSTM is a variant of Recurrent Neural Networks (RNNs) that is adept at solving long-term dependency problems. In a RNN model, the current state h_t is affected by the previous state h_{t-1} and the current input x_t, $h_t = \sigma(Wx_t + Uh_{t-1} + b)$, where σ is an activation function, and W, U, b are learnable parameters. In LSTM, f_t, i_t, o_t represent forget, input, and output gates, respectively. The gate utilizes the sigmoid

function σ to make the output value between $(0, 1)$, representing a certain proportion of historical information passing through.

$$\begin{aligned}
i_t &= \sigma(W_i x_t + U_i h_{t-1} + b_i) && \text{Input gate} \\
f_t &= \sigma(W_f x_t + U_f h_{t-1} + b_f) && \text{Forget gate} \\
o_t &= \sigma(W_o x_t + U_o h_{t-1} + b_o) && \text{Output gate} \\
\widetilde{c_t} &= \tanh(W_c x_t + U_c h_{t-1} + b_c) && \text{Candidate memory} \\
c_t &= f_t \cdot c_{t-1} + i_t \cdot \widetilde{c_t} && \text{Current memory} \\
h_t &= o_t \tanh(c_t) && \text{Current hidden state}
\end{aligned} \tag{2}$$

Our deep learning-based Granger causality test has advantages compared with the classical test: (1) The limitation of classical Granger causality, which can only analyze linear relationships, is overcome by using deep neural networks to model complex nonlinear relationships, and no prior knowledge is necessary; (2) By using joint modeling, the spatial complexity of the model can be reduced from $O(n^2)$ in existing methods to $O(n)$ while analyzing the causal relationships of multiple time series; (3) The method can add correlation time periods on the basis of the original Granger causality, enabling it to effectively analyze the causal relationships that dynamically change over time in complex systems.

As shown in Figure S4, our method outperforms the classical test method in the performance of Granger causality analysis on complex nonlinear multivariate time series data: The Granger causality between multivariate time series presents a circular form, and each time series can cause or be caused by any sequence (We set p=10, F=10, and sampling rate in Lorenz-96 to generate nonlinear data with 10 variables and 1000 consecutive time points for each variable). The ground truth of the Granger causality in generated multivariate time series is shown in Figure S4 middle, the Granger causality inferred by the classical method is shown in the left, and the Granger causality inferred by our method is shown in the right. The red box indicates incorrect inference. Our method is approximately 10% more accurate than the classical Granger causality methods.

Meanwhile, as shown in Table S5, When we use our MLP model to forecast strains, the result of inputting flight parameters inferred by the deep Learning-based Granger causality method outperforms that of inputting flight parameters inferred by the classical Granger causality method.

As shown in Table S4, more than 80% (257/308) pairs have deep learning-based Granger causality. Thus, using flight parameters to predict strains has potential.

S1.4 Data Preprocessing

Some effects of data processing can be seen in Figure S6.

S1.4.1 Data Correction

We first filter the time series of flight parameters and strains with 8Hz stopband cut-off frequency. The identified short period corruptions were classified as spikes, multi spikes, spike-step transitions, steps, hesitant steps, step reversals, dropouts,

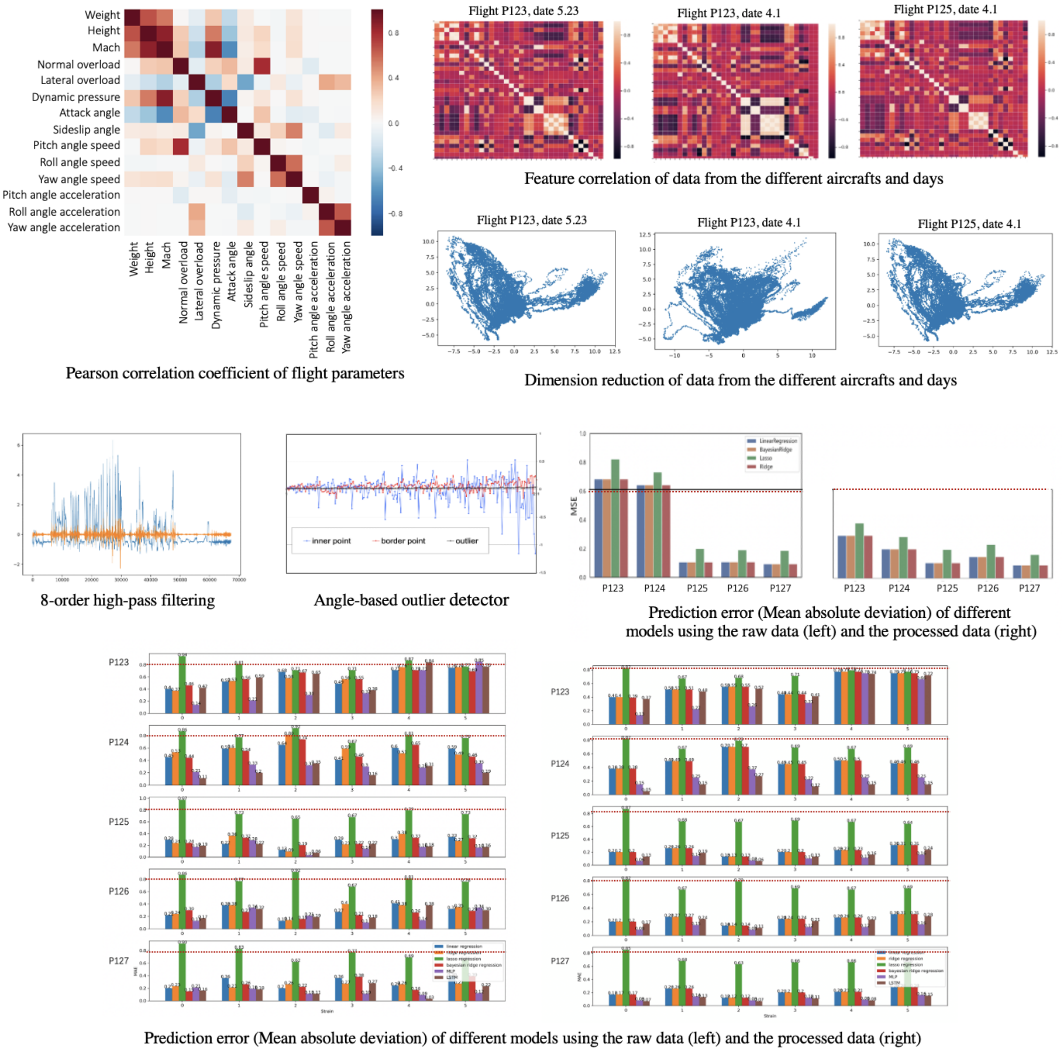

Fig. S5 Some Results in Data Preprocessing

DC signals, complex corruptions and jumps, shown in Figure 2. The identified long period corruptions are caused by temporarily or persistently inoperative sensors and calibration problems.

S1.4.2 Data Cleaning

Angle-Based Outlier Detector (ABOD) [2] considers the relations between each point and its adjacent points, the weighted cosine fraction and the variance of all adjacent points can be regarded as deviation scores. In dataset $\mathcal{D} \subseteq \mathbb{R}^d$, for a point $\vec{A}$ with a norm $.: \mathbb{R}^d \rightarrow \mathbb{R}_0^+$, the outlierness score is Equation 3, where $< .,. >$ denotes

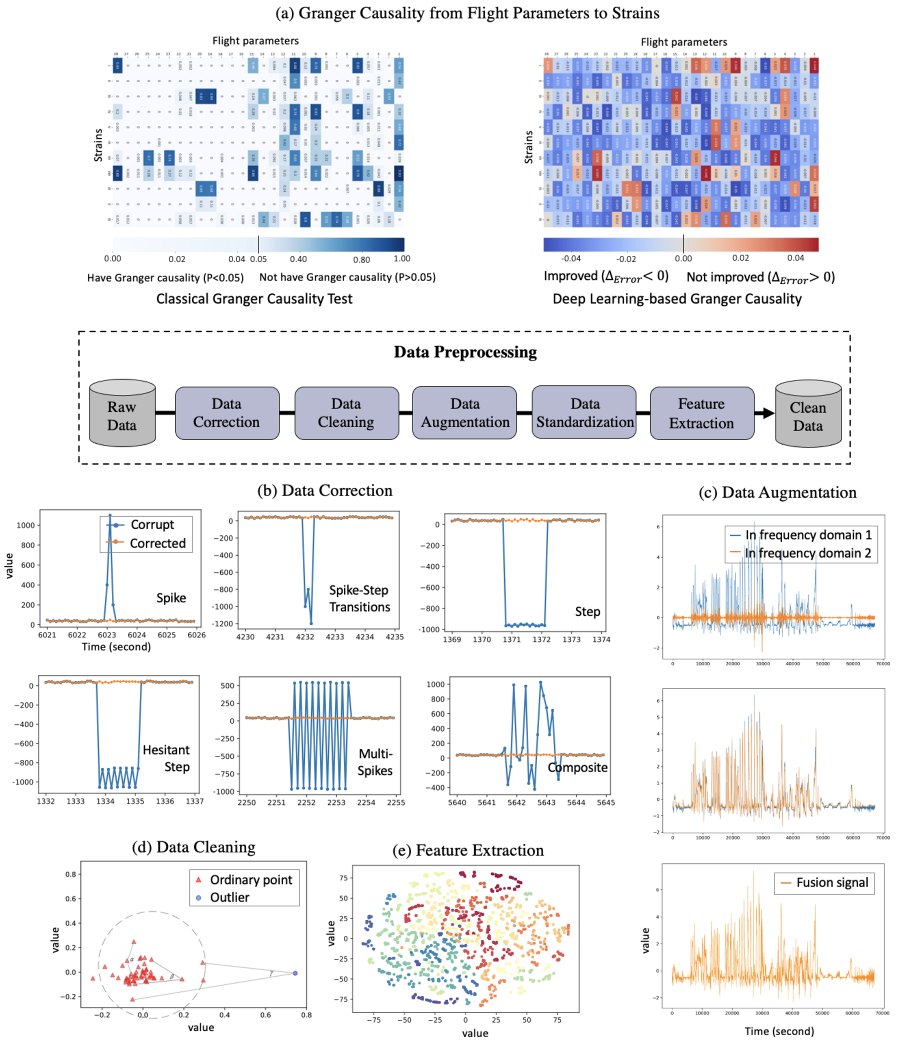

Fig. S6 Data Preprocessing

the scalar product $\mathbb{R}^d \times \mathbb{R}^d \rightarrow \mathbb{R}$ and $\overline{BC}$ denotes the difference vector $\vec{C} - \vec{B}$. As shown in Figure 2, compared with other points, if the angular variance of a point is quite small, it is likely to be an outlier. We set the ratio of outliers to 0.1%. About 20-100 outliers can be taken out in each flight test and will be picked out for subsequent analysis, as shown in Figure S5.

$$ABOD(\vec{A}) = VAR_{\vec{B},\vec{C}\in\mathcal{D}}\left(\frac{<\overline{AB},\overline{AC}>}{\overline{AB}^2\overline{AB}^2}\right) \tag{3}$$

S1.4.3 Feature Correlation Analysis

We use the Pearson correlation coefficient in Equation 4, where $\rho(A, B)$ is the correlation of sample A and B, *cov* is the covariance, and σ is the standard deviation. Some findings can be obtained through correlation analysis as shown in Figure S5: (1) Flight parameters have positive correlation (e.g. attack angle and dynamic pressure, pitch angle speed and normal overload), negative correlation (e.g. height and Mach, sideslip angle and lateral overload) and non correlation (e.g. weight and yaw angle acceleration, height and roll angle acceleration). (2) Compared with aircrafts, the dependence between flight parameters is more related to date / environment of the flight test. This may be related to the different actions of the aircraft in different flight tests. The standard variance of the correlation coefficient matrix for 34 days is 0.0125, while that for 5 aircrafts is 0.00574. Therefore, the correlation of flight data among different aircrafts changes little, and that among different dates changes greatly. Meanwhile, another correlation analysis, principal component analysis (PCA), can also show this finding. we use PCA to reduce 30 dimensional flight parameter to 2 dimensions and give visualization. The standard variance of distributions after dimensionality reduction for 34 days data is 26.980, while that for 5 aircrafts is 6.399.

$$\rho(A, B) = \frac{cov(A, B)}{\sigma A \sigma B} \tag{4}$$

S1.4.4 Feature Extraction

Fusing highly correlated features into one feature can eliminate the redundant information between features. The fused features have statistical uncorrelation, and can increase the amount of feature information. We use method, Multicollinearity analysis (If there is correlation between two or more variables in the model, it is called multicollinearity $X_i = \sum \theta_n X_n$), to drop the repetitive features with high correlation and retain the representative features. We use the Rigde estimation to calculate the multicollinearity between X_i and some other $X \subseteq \{X_n\}_{n=1}^{30}$. The multicollinearity θ_i is the solution obtained by Rigde estimation method in Equation 5, where k is an optional rigde parameter.

$$\theta_i = (X^T X + kI)^{-1} X^T X_i \tag{5}$$

Because the Deep Neural Networks (DNNs) have stronger learning ability under more features and data, finally, we just drop 2 features. Besides, we also extend features to multiply every two features as DNNs lack of abilities to learn multiplicative relation. Thus, the number of input feature is from 28 to $C_{28}^2 + 28 = 406$. We use some classical machine learning models, including Linear Regression (LR), Ridge Regression (RR), Lasso, Multi-Layer Perceptron (MLP), Long Short-Term Memory (LSTM), to test the data before and after data preprocessing. The prediction error, evaluated by Mean Absolute Deviation (MAE) in Equation 6, becomes smaller by using the processed data. The prediction accuracy using RR, LightGBM and MLP is summarized in Table S7, S8 and S9. It shows that data expansion has a great positive impact on DNN, while filtering has the opposite effect. This also proves our above

conclusion.

$$MAE = \frac{1}{N}\sum_{n=1}^{N}|E_n - \hat{E}_n| \tag{6}$$

Table S7 Prediction Accuracy of RR Using Raw Data, Filtering Data and Extended Data

Data \ Aircraft	P123	P124	P125	P126	P127
Raw data	75.39%	**86.21%**	85.11%	86.13%	88.91%
Filtering data	76.30%	84.28%	**86.29%**	**86.56%**	**91.25%**
Extended data	**84.31%**	85.00%	83.75%	86.40%	90.31%

Table S8 Prediction Accuracy of LightGBM Using Raw Data, Filtering Data and Extended Data

Data \ Aircraft	P123	P124	P125	P126	P127
Raw data	80.39%	88.44%	88.71%	88.33%	92.17%
**Filtering data**	81.39%	**88.98%**	**89.01%**	**89.92%**	**92.25%**
Extended data	**87.23%**	88.92%	88.66%	89.43%	92.01%

Table S9 Prediction Accuracy of MLP Using Raw Data, Filtering Data and Extended Data

Data \ Aircraft	P123	P124	P125	P126	P127
Raw data	86.21%	94.58%	93.71%	95.05%	96.44%
Filtering data	84.31%	94.00%	93.70%	93.47%	95.19%
**Extended data**	**92.11%**	**94.32%**	**92.93%**	**96.98%**	**97.01%**

S1.5 Multi-model Achitecture

We first divide the overall dataset into many subsets by some rules, then train different models for each subset. The combination of all sub-model is our multi-model. The main sub-model is based on deep neural networks, the optimal models are linear regression and decision tree, which will be introduced in the following sections.

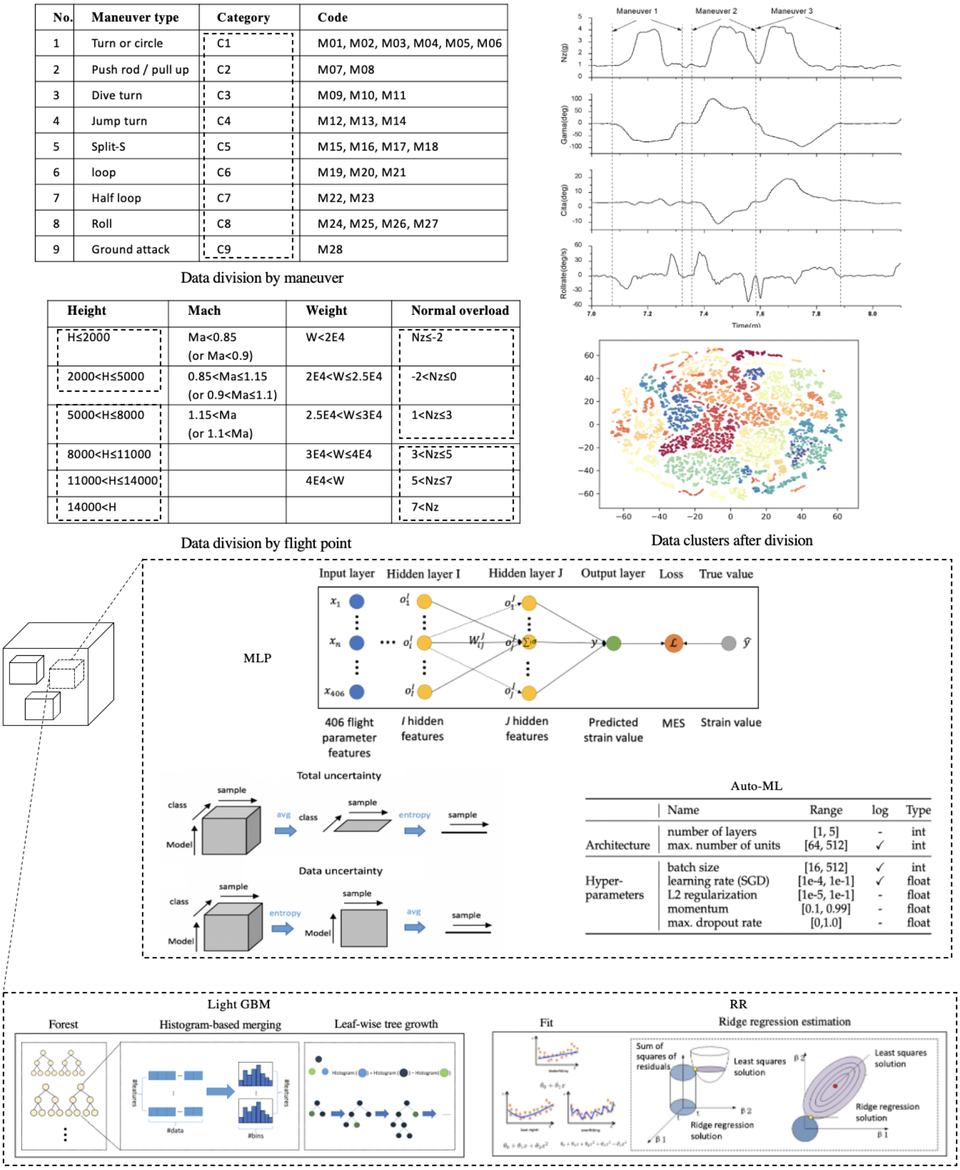

Fig. S7 Division Methods and Base Models in Multi-model

S1.5.1 Data Division Methods

We divide the dataset by two rules about flight parameters: Maneuver based division and PITS based division.

Maneuver-based method divides data according aircraft attitude. Before maneuver identification, all data need to be preprocessed, including deleting the invalid take-off and landing, eliminate the data points beyond the error code limit, filter the

Table S10 Classification of Maneuvers

S/N	Basic-maneuver	Sub-maneuver	Code
C1	Turn	Common turn	M01
		Sustained turn	M02
		Decelerated turn	M03
		Steep left and right turn	M04
		Continuous steep turn	M05
	S turn		M06
C2	Pull/Push	Pull	M07
		Push	M08
C3	Diving turn	20° diving and turn	M09
		30° diving and turn	M10
		45-60° diving and turn, steep dive and turn	M11
C4	Pull-up turn	20° pull-up and turn	M12
		30° pull-up and turn	M13
		45 60° pull-up and turn, steep pull-up and turn	M14
C5	Split-s	Split-s, oblique split-s	M15
		Split-s with vertical half roll	M16
		Oblique split-s with vertical half roll	M17
		High-speed of split-s with vertical half roll	M18
C6	Loop	Complete loop	M19
		Complete oblique loop	M20
		Oblique-loop turn	M21
C7	Half-loop	Half-loop overturn	M22
		Half-loop overturn with vertical half roll	M23
C8	Roll	Dual pull-up turns	M24
		45-60° pull-up and roll	M25
		30-45° diving and roll	M26
		Slow level roll, larger-radius roll	M27
C9	Air-to-ground attack	Divie-attack-pull	M28

overload in the normal direction of the center of gravity, etc. We define that the aircraft starts from $1g$ level flight state, performs a series of actions, and then returns to $1g$ level flight state, which is a complete maneuver. At the peak point of normal overload, we search forward and backward along the time axis respectively. The first time point at which the normal overload is about $1g$ and the aircraft state parameters such as roll angle, pitch angle and roll angle speed are near 0 is regarded as the start time and end time of maneuver. According to this principle, various maneuvers can be extracted in the form of multi parameter time history. The example in top right of

Figure S7 illustrates the extraction of maneuver history from flight parameters. Referring to the flight training program, intensity specification, aircraft operation manual and analyzing a large number of measured maneuver history data, this paper summarizes 28 common maneuver type in 9 categories as shown in the first table in Figure S7 and Table S10, such as turn or circle, dive turn and loop. Each maneuver has its code, such as M01, M02 and M03.

PITS-based method divides data according to different value range. As shown in the second table in Figure S7, we selected 4 representative flight parameters to divide the data. According to height, the dataset can be divided into 6 sets; According to Mach, the dataset can be divided into 3 sets; According to weight, the dataset can be divided into 5 sets; According to normal overload, the dataset can be divided into 6 sets. Thus there are up to $6 \times 3 \times 5 \times 6 = 540$ subsets.

We first divide the data with maneuver, and then divide the obtained subset with PITS. Up to $28 \times 540 = 15,120$ data sets can be obtained by this method. In order to avoid too small sample size, in practice, we use 9 maneuver categories and $2 \times 2 = 4$ PITS sets. The overall subsets are up to $9 \times 4 = 36$ as shown in dashed box.

S1.5.2 Deep Neural Networks

Mathematical theory proves that the three-layer neural network can approach any nonlinear continuous function with arbitrary accuracy. Therefore, neural networks are suitable for solving problems with complex internal mechanism. i.e., they have the potential to accurately model the highly nonlinear relationship between flight parameters and strains.

Multi-Layer Perceptron (MLP) is a neural network model suitable for general regression tasks. The structure of MLP model is shown in Figure S7. A classical MLP contains input layer, hidden layer and output layer. States are transferred by weighting between adjacent layers. Nonlinear activation occurs on neurons in the hidden layer. For example, the state o_j^J in J-th hidden layer is the transformation of all states $\{o_i^I\}_{i=1}^I$ in I-th hidden layer:

$$o_j^I = \sigma(\sum_{i=1}^{I} W_{ij} o_i^I + b_{ij}) \tag{7}$$

If the input is X, the output will be $f(X)$ after the nonlinear mapping of model f. Deep neural networks are updated by gradient descent algorithm, where the minimum value is solved along the descending direction of the gradient. We use Mean Square Error (MSE) as the minimum objective function, i.e., the loos function in Equation 8. The weight is updated by Equation 9, where α is the learning rate.

$$\mathcal{L}_{MLP} = \sum (E - f(X))^2 + \lambda \|W\|_2^2 \tag{8}$$

$$W_{updated} = W - \alpha \frac{\partial \mathcal{L}_{MLP}}{\partial W} \tag{9}$$

In the training process, we integrate some optimization methods of feedback mechanism, model uncertainty evaluation, neural architecture search, parameter update strategy, etc.

The hyper-parameters are searched by Automatic Machine Learning method (Auto-ML) to improve the fitting effect of the model. The optimized hyper-parameters in Auto-ML include structural hyper-parameters and training hyper-parameters. Based on the structure of MLP, the search space composed of 7 hyper-parameters is considered, of which 5 are the training hyper-parameters commonly used in momentum SGD training. The search space is shown in Figure S7.

The model uncertainty is defined to improve the robustness. Generally, the overall uncertainty in the task may come from two sources: one is caused by over-fitting and non-fitting of training, incorrect learning rate and different hyper-parameter settings; the other is caused by data noise and label ambiguity. From this perspective, the overall uncertainty can be divided into the model uncertainty and the data uncertainty:

$$\mathcal{H}[\mathbb{E}_{P(\theta|\mathcal{D})}[P(y|x^*,\theta)]] = \mathcal{I}[y,\theta|x^*,\mathcal{D}] + \mathbb{E}_{P(\theta|\mathcal{D})}[\mathcal{H}[P(y|x^*,\theta)]] \tag{10}$$

We minimize the model uncertainty by $U_{model} = U_{total} - U_{data}$ according to Equation 10. In detail, we combine Monte-Carlo Dropout (MC-Dropout) mechanism to evaluate and optimize the model uncertainty: Using dropout mechanism to train the model; Testing the model and obtain the prediction matrix (# model, # sample, # prediction). The overall uncertainty is: the cross entropy of the average prediction results of all models on the sample; The data uncertainty is the average prediction result of the cross entropy of the sample on all models. The uncertainty of the model is: overall uncertainty - data uncertainty.

We use mean square error L_{mse} and model uncertainty loss $L_{\text{uncertainty}}$ as the minimum bi-objective $\mathcal{L}_{\text{MLP}}$ (Equation 11), where γ_1, γ_2 are weight coefficients, f is MLP model, $\theta, \mathcal{D}$ are model parameters and parameter distribution, $\mathcal{H}$ is the entropy of the predictive distribution.

$$\begin{aligned} \mathcal{L}_{\text{MLP}} &= \gamma_1 \mathcal{L}_{\text{mse}} + \gamma_2 \mathcal{L}_{\text{uncertainty}} \\ L_{\text{mse}} &= \sum (E - f(X))^2 + \lambda \|W\|_2^2 \\ L_{\text{uncertainty}} &= \underbrace{\mathcal{H}[\mathbb{E}_{P(\theta|\mathcal{D})}[P(f(X)|X,\theta)]]}_{\text{Total Uncertainty}} - \underbrace{\mathbb{E}_{P(\theta|\mathcal{D})}[\mathcal{H}[P(f(X)|X,\theta)]]}_{\text{Data Uncertainty}} \end{aligned} \tag{11}$$

S1.5.3 Non Deep Learning Models

Ridge Regression (RR) constructs the linear relationship between independent variables X and dependent variables Y in Equation 12. β is the regression coefficient and μ is the random error. RR uses the maximum posterior probability based on Gaussian prior distribution. As shown in Figure S7, RR can reduce the regression coefficient

Table S11 Prediction Accuracy of Different Models

Model \ Aircraft	P123	P124	P125	P126	P127
RR	84.31%	85.00%	83.75%	86.40%	90.31%
LightGBM	87.23%	88.92%	88.66%	89.43%	92.01%
MLP	92.11%	94.32%	92.93%	96.98%	97.01%
Multi-RR	90.15%	90.12%	86.90%	89.75%	92.14%
Multi-LightGBM	92.01%	93.94%	93.03%	94.46%	96.99%
Multi-MLP	94.21%	96.52%	95.43%	97.62%	98.04%
**Multi-model**	**95.10%**	**96.93%**	**96.07%**	**98.00%**	**98.36%**

and make the model relatively stable.

$$Y = \beta_0 + \beta_1 x_1 + ... + \beta_n x_n + \mu \tag{12}$$

Gradient Boosting Machine (LightGBM) [3] fits the step function between partitions based on a series of if-else rules in Equation 13. They recursively form a subtree G_c according to condition c and return the function f_t at the leaf node. LightGBM are based on histogram algorithm and uses the negative gradient of the loss function as the residual approximation of the current decision tree to fit the new decision tree.

$$Y = \sum_c if(b(x) == c) * G_c(X) \textit{ end with } G_c(X) = f_t(X) \tag{13}$$

The loss functions of RR and LightGBM are in Equation 14,15. In addition to the squared error term, they all have the regularization term to avoid overfitting and model complexity.

$$\mathcal{L}_{RR} = \sum (E - \beta X)^2 + \lambda \|\beta\|_2^2 \tag{14}$$

$$\begin{aligned} \mathcal{L}^t_{LightGBM} &= \sum (E - \hat{E}^t)^2 + \sum_{k=1}^{t} \Omega(f_k) \\ \hat{E}^t = \hat{E}^{t-1} + f_t(X), &\quad \Omega(f_k) = \gamma T + \lambda \|W_t\|_2^2 \end{aligned} \tag{15}$$

S1.5.4 Prediction Accuracy

As shown in Table S11 and Figure S8, the multi-model achieves the best accuracy. As shown in Figure S9, using the dual objective as the loss function can increase the stability and generalization ability of our model. And the trade-off between the two objective is set at 1:1 (the coefficient of $L_{\text{mes}} = 0.5$).

S1.6 Coefficient Calibration

We only implement the ground load calibration for the reference measured-load aircraft 0 to get its strain-load equation in Equation 16. Then we calibrate the strain

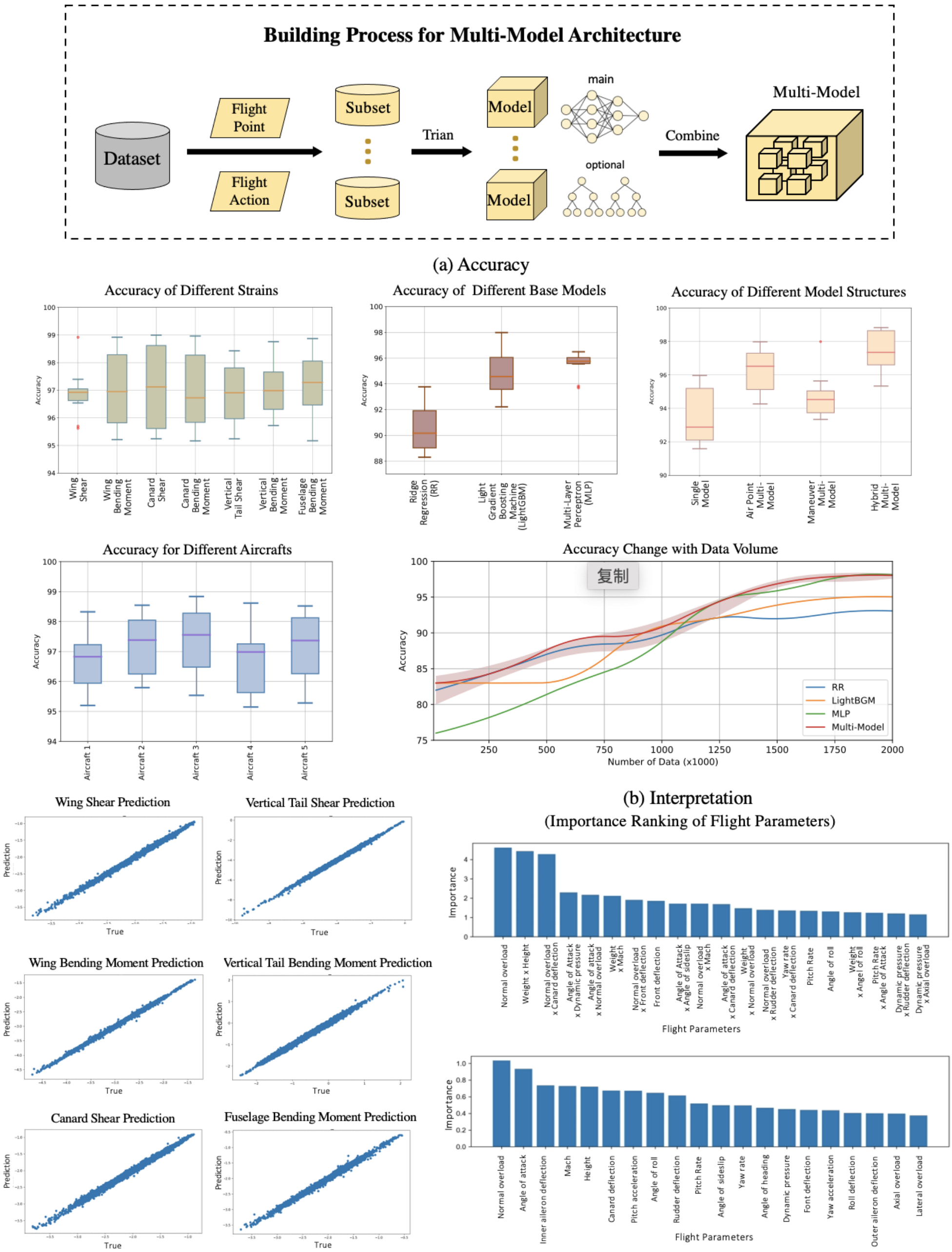

Fig. S8 Prediction Accuracy

coefficients between aircraft a and the reference measured-load aircraft 0 and create their relation in Equation 17. Finally, we can get the strain-load equation for all

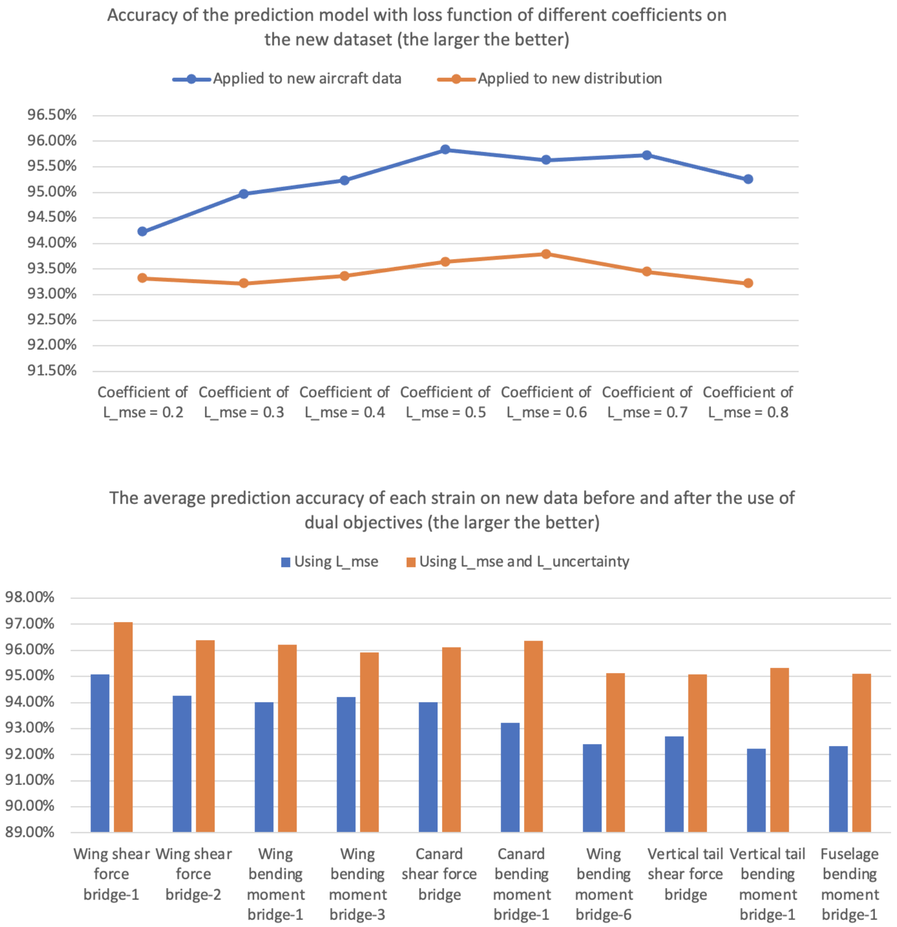

Fig. S9 Model Performance when Using the Dual Objective as the Loss Function

aircraft in a fleet by Equation 18.

$$F = \sum_i k_i^0 \cdot E_i^0 + b^0 \tag{16}$$

$$E^0 = SF^a E^a + b^a \tag{17}$$

$$F = \sum_i k_i^0 \cdot (SF_i^a E_i^a + b_i^a) + b^0 = \sum_i \alpha_i E_i^a + B \tag{18}$$

We assume that the coefficient between the strain E_i^0 of reference measured-load aircraft 0 and the strain E_i^a of the aircraft a is SF^a. Before calibrating the value of k, we need to build the corresponding strain pairs (E_i^0, E_i^a). There are two strategies to find the strain pair as shown in Figure S10.

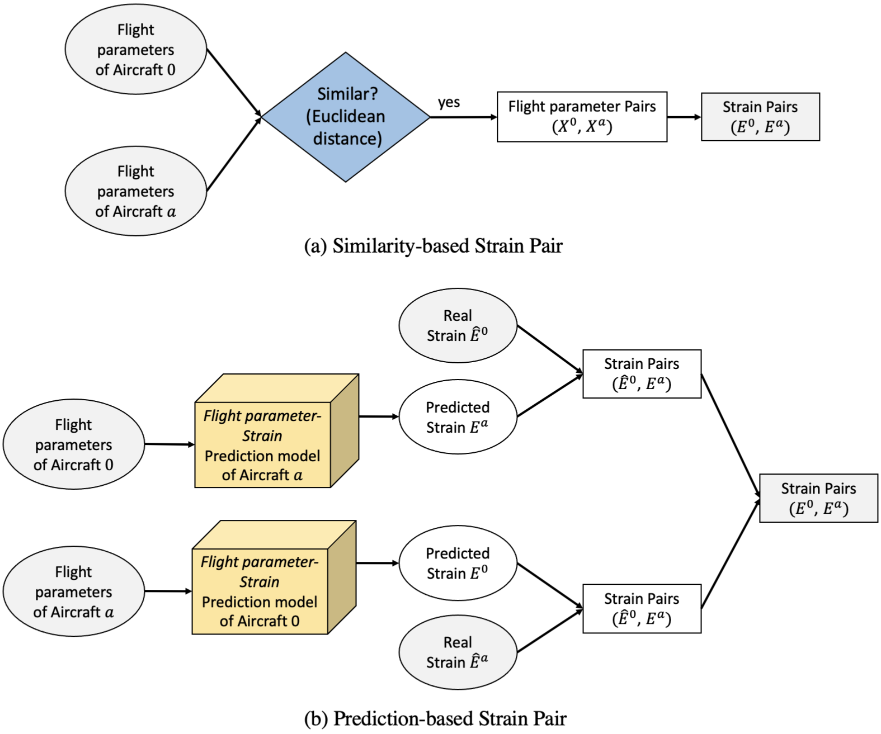

Fig. S10 Two Strategies to Find the Strain Pair

S1.6.1 Similarity-based Strain Pairs

For the dataset of flight parameters $\{X_i^0\}_{i=1}^{N_0}$ of aircraft 0 and $\{X_j^a\}_{j=1}^{N_a}$ of aircraft a, we calculate the Euclidean distance $ED_{X_i^0,X_j^a}$ of X_i^0 and X_j^a. For one X_i^0, we will calculate the Euclidean distance N^a times, and pick X_j^a with the minimum ES to form the data pair (X_i^0, X_j^a). For $\{X_i^0\}_{i=1}^{N_0}$, there are $N^0 \times N^a$ times calculations. According to strains corresponding to flight parameters, we can get (E_i^0, E_j^a). In the same way, for one X_j^a, we will calculate the Euclidean distance N^0 times, and there are $N^0 \times N^a$ times calculations. Thus, the complexity of similarity-based strategy is $\mathcal{O}(n^2)$, where n is the number of samples and usually millions. And due to the different among data distribution of aircraft, the direct searching based on the similarity could cause the data migration.

$$ED_{X_i^0,X_j^a} = \sqrt{(X_i^0 - X_j^a)^2} \tag{19}$$

S1.6.2 Prediction-based Strain Pairs

In order to reduce the algorithm complexity and avoid pairing offset, we propose a novel data pair construction method based on the prediction model. As shown in the blue box of Figure 1, we use the model of the aircraft 0 to predict the strain E^0 from

flight parameters of aircraft a. Combining with the corresponding real strain $\hat{E}^a$ of aircraft a, we can get pair $(E^0, \hat{E}^a)$. Then we also use the model of aircraft a to predict the strain E^a from flight parameters of aircraft 0 and get $(\hat{E}^0, E^a)$. Finally, we integrate them to get the pair dataset (E^0, E^a). The complexity will be reduced from $\mathcal{O}(n^2)$ to $\mathcal{O}(n)$.

$$E^a = f^a(X^0), \quad E^0 = f^0(X^a) \tag{20}$$

Table S12 Calibrated Coefficients k between 10 Strains of 5 aircrafts

k	P124	P125	P126	P127
P123	0.98 0.95 1.00 0.98 0.81 1.03 0.96 0.87 0.89 0.87	0.91 0.82 0.92 0.92 0.80 0.85 0.82 1.17 1.14 0.80	0.80 0.87 0.84 0.96 0.81 0.86 0.85 1.16 1.08 0.80	0.85 0.80 0.83 0.90 0.80 0.84 0.78 1.16 1.01 0.87
P124		0.84 0.89 0.90 0.80 1.07 0.90 0.88 0.84 0.89 0.95	0.92 0.99 0.86 0.97 1.02 0.92 0.95 0.82 0.81 1.19	0.81 0.82 0.83 0.83 1.12 0.85 0.77 0.85 0.98 0.93
P125			1.11 1.05 0.91 1.05 1.08 1.00 0.96 1.00 0.99 0.80	0.76 0.89 0.82 0.92 0.86 0.84 1.05 0.75 0.89 0.83
P126				0.87 0.81 0.83 0.88 0.88 0.78 0.95 0.80 0.91 0.97

Table S13 Calibration R^2 between 10 Strains of 5 aircrafts

R^2	P124	P125	P126	P127
P123	0.99903 0.99961 0.99902 0.99941 0.93842 0.99927 0.99915 0.97335 0.92634 0.96965	0.99868 0.99960 0.99920 0.99936 0.99868 0.99914 0.99907 0.96843 0.93966 0.98161	0.99839 0.99959 0.99941 0.99922 0.99864 0.99939 0.99921 0.94734 0.92315 0.97581	0.99731 0.99848 0.99824 0.99885 0.99782 0.99905 0.99892 0.97545 0.92423 0.97099
P124		0.99864 0.99960 0.99902 0.99916 0.98526 0.99906 0.99924 0.99887 0.99885 0.99336	0.99970 0.99982 0.99977 0.99981 0.98187 0.99904 0.99943 0.99871 0.99432 0.99901	0.99961 0.99967 0.99974 0.99919 0.98422 0.99899 0.99910 0.99814 0.99787 0.99241
P125			0.99980 0.99976 0.99990 0.99988 0.99988 0.99922 0.99932 0.99869 0.99882 0.99579	0.99873 0.99941 0.99986 0.99941 0.99981 0.99903 0.99895 0.99842 0.99851 0.99718
P126				0.99979 0.99978 0.99974 0.99927 0.99983 0.99888 0.99910 0.99897 0.99867 0.99676

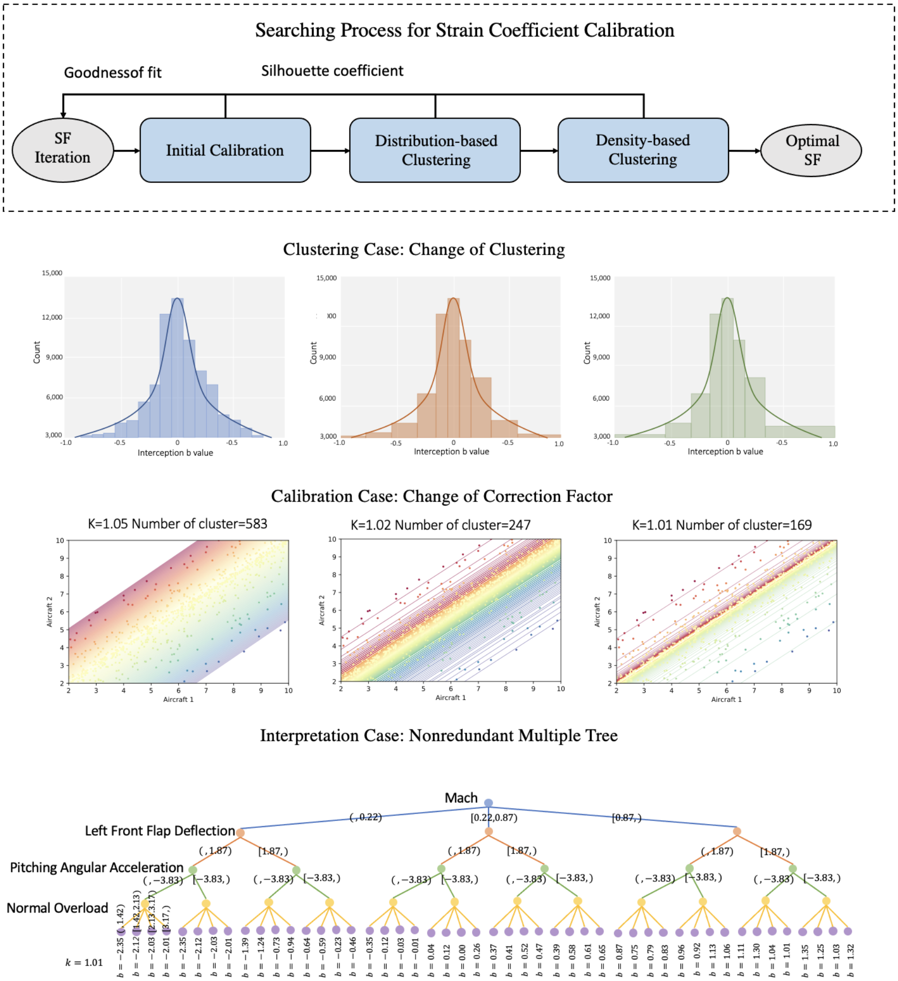

Fig. S11 Calibration Process

After getting strain pairs (E^0, E^a), we then calibrate their coefficient SF^a. For one kind of strain, there are M pairs $\{(E^0, E^a)_m\}_{m=1}^{M}$. We assume that for one kind of strain, all strain pairs $(E^0, E^a)_m$ have the same coefficient SF^a but different intercept b^a.

For the correction factor SF, we design an iterative and feedback process as shown in Figure 4. Through iterating the feasible space of SF, the intercept b under the current SF is clustered based on the distribution-based method and the density-based method. At the same time, the clustering silhouette coefficient S and the coefficient of determination R^2 are obtained and feedback to continue to iterate and adjust SF. S evaluates the clustering performance and R^2 evaluates the goodness of fit.

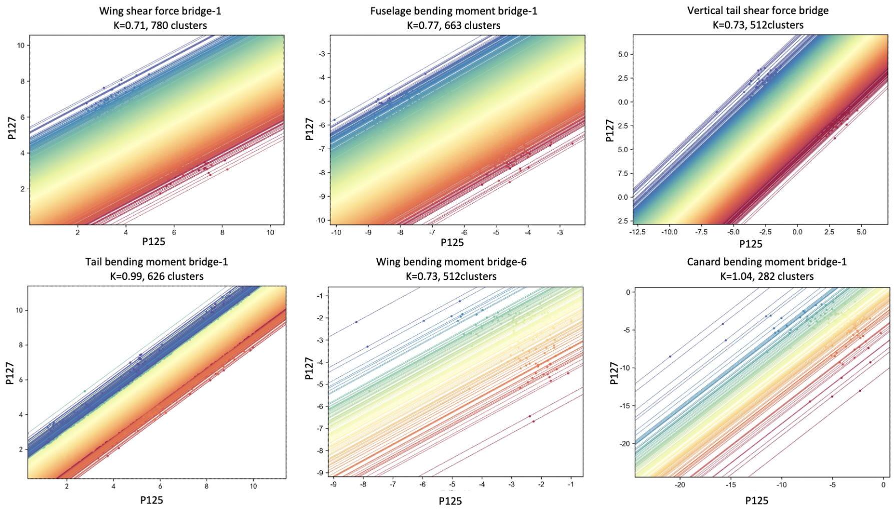

Fig. S12 Calibration for 6 Strains between Aircraft P125 and Aircraft P127

S1.6.3 Distribution-based Clustering

It models b as a Gaussian distribution $\mathcal{N}(\mu, \sigma^2)$ and divides the σ interval equally and get clusters.

S1.6.4 Density-based Clustering

It merges clusters with small sample, especially that at the edge of the distribution. It is based on Density-Based Spatial Clustering of Applications with Noise (DBSCAN) [4]. It divides the region with high enough density into clusters, and can find clusters of arbitrary shape.

The silhouette coefficient S in Equation 21 evaluates the clustering performance by calculating the intra cluster distance and inter cluster distance. C_b is points in the same cluster as b, C_b is points in different clusters from b. D is The Euclidean distance. The coefficient of determination in Equation 22 evaluates the goodness of fit by calculating the total sum of square between true strain $\hat{E}$ and their average $\overline{E}$, the explained sum of squares between the predicted value E and $\overline{E}$. It shows the fitting degree of regression line determined by SF and b to observed strain pair of E^0 and E^j. Trough the process of updating SF, clustering, calculating S and R^2, feedback, we finally determine the optimal SF.

$$
\begin{gathered}
S = \frac{1}{|b|} \sum_{b} \frac{D_{intra} - D_{inter}}{Max\, \{D_{intra}, D_{inter}\}} \\
D_{intra} = Min\ Avg\ D(b, C_{\sim b}), \quad D_{inter} = Avg\ D(b, C_b)
\end{gathered}
\tag{21}
$$

$$
R^2 = 1 - \frac{\sum_n (E_n - \overline{E})^2}{\sum_n (\hat{E}_n - \overline{E})^2} \tag{22}
$$

The calibrated coefficient for 10 strains (Wing shear bridge-1, Wing shear bridge-2, Wing bending moment bridge-1, Wing bending moment bridge-3, Wing bending moment bridge-6, Canard shear bridge, Canard bending moment bridge-1, Vertical tail shear bridge, Vertical tail bending moment bridge-1, Fuselage bending moment bridge-1) of each two aircrafts (P123, P124, P125, P126, P127) is shown Table S12. All coefficients are in the range of $0.8 - 1.2$. Their values of goodness of fit are in Table S13. Most R^2 are higher than 0.99.

S1.7 Interpretation Methods

S1.7.1 Interpretation for Multi-model

As the neural network model is complex and naturally unexplainable, we interpret its results by the key features of model perception, not the model itself. The interpretation is flight parameters that have a great impact on the strain prediction results. For MLP, we use the SHapley Additive explanation (SHAP) method [5]. Each kind of flight parameter is calculated to get a Shapley value of Equation 23, which is the average contribution of a feature to the prediction in all possible coalitions. LightGBM and RR are inherently interpretable through the information gain Gain and the independent variable coefficient β. Ent is the information entropy, D^v is a subset divided by x_i. According to ranking the features of the Shapley value, information gain and independent variable coefficient, we can get the important flight parameters when predicting strains.

$$\Phi_i(f, x_i) = \sum_{z \subseteq X \backslash \{x_i\}} \frac{|z|!(M - |z| - 1)}{M!} \cdot [f(x \cup x_i) - f(z)] \tag{23}$$

$$\text{Gain}(D, x_i) = \text{Ent}(D) - \sum_{v} \frac{|D^v|}{|D|} \text{Ent}(D^v) \tag{24}$$

Through this method, we can get the flight parameters that are important for strain prediction. Table S14 and Figure S13 show the importance of all flight parameters. For example, the five most important flight parameters are normal overload, angle of attack, inner aileron deflection, Mach and barometric altitude (height). The acquisition of important flight parameters is of great significance to verify the method in physical meaning and find potential and new physical relations.

S1.7.2 Interpretation for Calibration

We design the alternative model based method to explain how to determine the specific b under the current calibration with SF. We propose a novel Nonredundant Multiple Tree as the alternative model. Under the same SF, the samples with the same intercept b are regarded as in the same class. We design a classification model to learn the relation between flight parameters and intercept classes. The decision tree is naturally interpretable. But it is usually the binary tree, and the same classification feature will appear in different layers, resulting in multiple paths that can reach the same class, which can not form a more intuitive explanation like the PITS division

Table S14 The Importance Ranking of Flight Parameters When Predicting Strains

No.	Flight parameter	Importance	No.	Flight parameter	Importance
1	Normal overload	0.98	16	Right flap deflection	0.42
2	Angle of attack	0.89	17	Yaw acceleration	0.42
3	Right inner aileron deflection	0.78	18	Roll deflection	0.40
4	Mach	0.78	19	Left outer aileron deflection	0.40
5	Height	0.77	20	Axial overload	0.40
6	Left Canard deflection	0.71	21	Lateral overload	0.38
7	Pitch acceleration	0.70	22	Left flap deflection	0.28
8	Right canard deflection	0.69	23	Right outer aileron deflection	0.26
9	Angle of roll	0.68	24	Left rudder deflection	0.26
10	Rudder deflection	0.66	25	Right rudder deflection	0.24
11	Pitch Rate	0.60	26	Right canard deflection	0.24
12	Angle of sideslip	0.46			
13	Yaw rate	0.45			
14	Angle of heading	0.45			
15	Dynamic pressure	0.44			

method. Thus we propose the Nonredundant Multiple Tree and prove that there is an equivalent Nonredundant Multiple Tree without information loss for the full binary tree generated by discretization of continuous features, merging and pruning.

The three traditional decision tree methods (ID3, C4.5 and CART) use information gain, information gain rate and Gini index to divide the features respectively. They are suitable for dealing with discrete features, while the flight parameters is a kind continuous data. Nonredundant Multiple Tree uses the dichotomy to discretize the characteristics of continuous flight parameters: Take the median point of all data as the candidate partition point; Calculate the information gain value IV for each partition point and select the optimal partition feature; The current node partition feature is continuous feature, which can be used as the partition feature of its descendant nodes.

As shown in Figure S14, in the traditional decision tree, each partition feature may appear in many layers, which limits the understanding of its semantic information and logical rules after visualization. In order to avoid features appearing in the root node and the descendant node, we prove that for the full binary tree generated by continuous features, there is an equivalent nonredundant multiple tree without information loss.

Assuming that the original tree is a full binary tree with N-layer continuous features, there are $2N-1$ nodes and at most $2N-1$ types of leaves. Suppose a total of K features are used, taking the maximum information entropy of decision features, and the number of bifurcation conditions of each feature is the same, then there are $\frac{2^{N-1}-1}{K+1}$ judgment intervals for each feature in the new tree. For the new tree, if the we extract the same features into one layer and form a nonredundant multiple tree having $K+1$ layers and $\frac{2^{N-1}+1}{K+1}$ branches in each layer, the final number of category leaves is $(\frac{2^{N-1}+1}{K+1})^k$. Therefore, in this study, we only need to satisfy $(\frac{2^{N-1}+1}{K+1})^k \geq 2^{N-1}$ to construct an equivalent nonredundant multiple tree. As shown in curve of $y=(\frac{15}{x}+1)^x-16$, When $N=5$, $k>1$ can satisfy $(\frac{15}{k}+1)^k-16 \geq 0$.

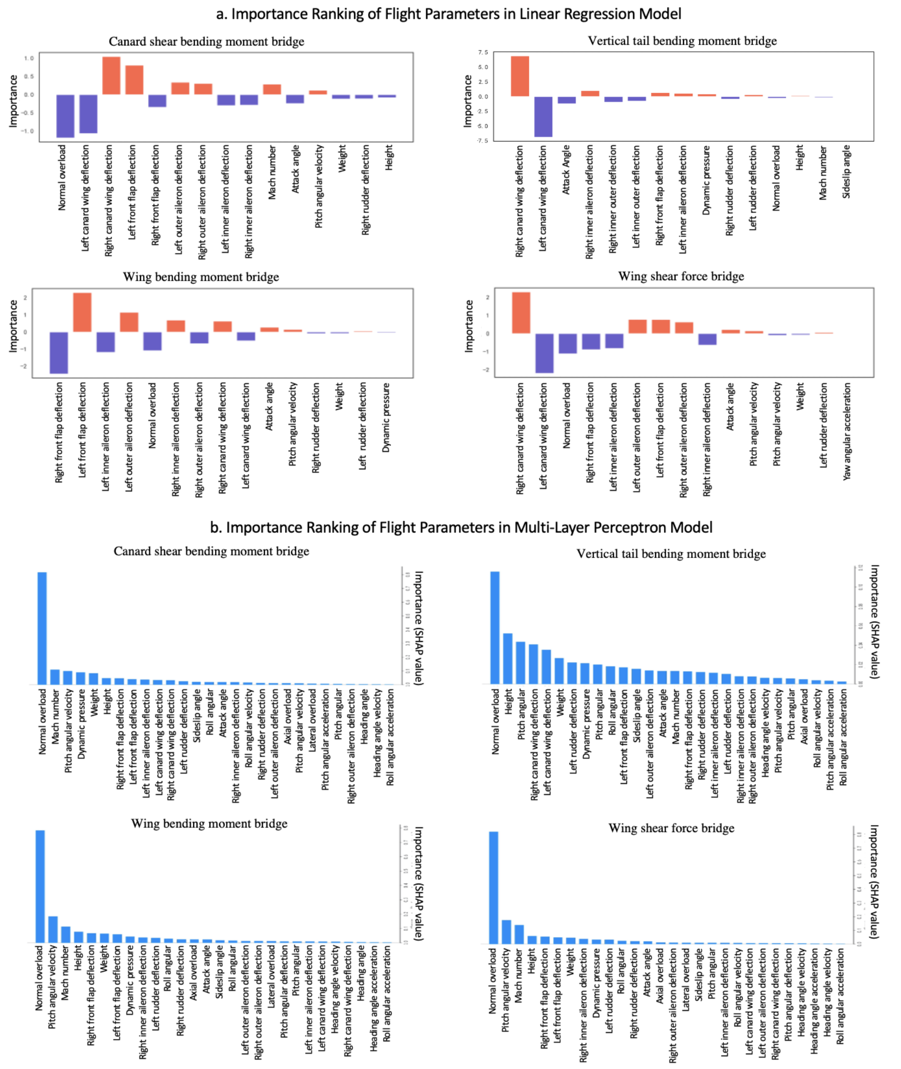

Fig. S13 The Importance of Flight Parameters for Stains

$$IV = -\sum_i p(v_i)logp(v_i) \tag{25}$$

The interpretation result is shown in Table S15. It is a air point (introduced in data division methods section) like form. 4 key classification characteristics are obtained: Mach, left flap deflection, pitching angular acceleration and normal overload.

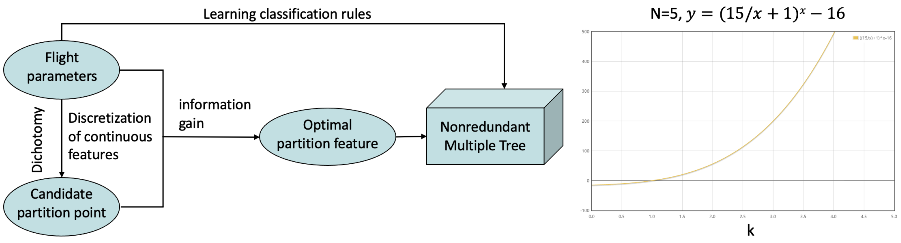

Fig. S14 Nonredundant Multiple Tree

Table S15 Intercept Division Rule When Calibrating Strains

	Mach	Pitch angular acceleration	Right outer aileron deflection	Normal overload
Range	$(, 0.22)$<br>$[0.22, 0.87)$<br>$[0.87,)$	$(, -1.87)$<br>$[-1.87,)$	$(, -3.83)$<br>$[-3.83,)$	$(, 1.42)$<br>$[1.42, 2.13)$<br>$[2.13, 3.17)$<br>$(3.17,)$

S1.8 Prototype System

The project is based on browser and server architecture. The browser is the interface of the user's operating system. The user makes a request to the server through the browser interface. The server provides data services, operates the data, and then returns the results to the system interface. Through the cooperation of the front and back ends, the system will show the data of the background server to the foreground users in the browser. Our Chinese version of the system is publicly available on https://github.com/SCXsunchenxi/LoadPrediction.

S2 Supplementary Discussion

Aircraft with high cost, complex structure and safety requirements must have high reliability [6]. Aircraft structural health monitoring is of great significance to prevent aircraft damage, avoid air crash and promote the development of aviation industry and military construction. With the maturity of safety and design theories of electrical appliances, accidents caused by the failure of aircraft electronic components are decreasing, while faults of aircraft structure system, especially fatigue failure, become increasingly prominent. Once fatigue damage occurs, it often leads to air crash and casualties [7]. The monitoring and management of structural load, deformation and stress run through the whole process of aircraft design, manufacturing, test and service.

Load overload is the main cause of aircraft fatigue failure. In flights from takeoff to landing, the aircraft structures are constantly subjected to alternating loads. Statistics indicate that the load is the major cause of the fatigue failure, accounting for more than 50% of the total failures of mechanical structures. The load-caused failure has high randomness and great risk as it has no obvious damage precursor or significant deformation. National military standard of the people's Republic of China

(GJB) stipulates that 'In all states that the flight control system may encounter, the body structure of the aircraft shall have sufficient structural integrity to bear the load caused by expected and possible faults'. Thus, obtaining the load history of aircraft structures is key to monitoring the structural damage and evaluating the aircraft's life.

The existing work and practices can be divided into three categories.

S2.1 Measurement-based method: from strains to load

In most classical practice, the aircraft load is calculated by directly measured strain and established strain-load equation [8, 9]. That is, researchers first implement load calibration test on the ground. In this process, the strain is collected by the installed (pasted) strain gauges on the main load-transferred path of aircraft structure. Then, they establish the Strain-Load quantitative relationship based on the multiple linear regression analysis.

However, the low availability of strain gauges decreases the accuracy and increases the cost of this method. The strain gauge has the risk of falling off, failure, data drift, and missing and the accuracy suffers from the accumulative effects of factors such as orientation precision, bonding, age, and environments [10]. The data error could be up to 40% when the operational demand is high [11].

In order to improve the measurement accuracy of strain gauges, U.S. Boeing Company designed FBG temperature sensor for A340-600 passenger aircraft [12]; Japan has designed a sensor that can obtain 17 strains with only 3 optical fibers [13], while the same number of traditional strain acquisition requires at least 34 cables; Canada has carried out axial strain research at 2371 positions in the direction of optical fiber on F/A-18 aircraft [14]; Israel has studied the condition based maintenance of helicopter composite blades by installing optical fiber sensors on the trailing edge of S-76 helicopter main blades [15]; The Dutch Aerospace Center has developed an integrated optical fiber sensing technology and traditional data acquisition equipment system to solve the problem of synchronization and correction of optical fiber sensing data and other flight test data [16].

Although many practices have improved the measurement accuracy, with the increasing complexity of aircraft structure, more strain gauges are required to be refitted and this method becomes more cumbersome [17]. The pasting process of strain gauges is complex and can not be done mechanically. Fitting, operating, and maintaining the system accuracy would require regular expensive calibration exercises and high labor costs, and once the strain gauge pasted inside the structure fails, it can hardly be compensated. So it is time-consuming and labor-intensive. In many practices, the cost of producing, pasting, and maintaining strain gauges accounts for about 80% of the total cost of structural health monitoring.

S2.2 Prediction-aided method: from flight parameters and strains to load

Compared with the strain data, the flight parameter data is more reliable, easy to obtain and low-cost [18]. Flight parameters are recorded by flight data recording systems, such as flight recorders, aircraft sensors and airborne systems. They represent flight status, attitude and the working status of each aircraft system.

Thus, in the current practice of most countries, in order to alleviate the excessive dependence on strains, researchers have implemented the prediction-aided method based on available flight parameters to calculate the load [19, 20, 21, 22, 23]. That is, some equations between flight parameters and load are established to avoid measuring some strains. Combined with the strain and the flight parameter, the U.S. F-22 aircraft has been established a total of 278 load calculation equations and stress calculation equations of more than 800 control points corresponding to the subsonic, transonic, supersonic states of the aircraft; European EF2000 aircraft is equipped with SHM system, and 17500 calculation templates are established by comparing and iterating with the template on the stress of each control point in the way of parametric monitoring [24]; The Japanese F-2 aircraft has been constructed 22 equations to calculate the values on the main force transmission components by multiple regression analysis technology [25].

However, in order to build the equations mentioned above, strength tests of aircraft structure, including whole fuselage static test, fatigue test, impact test, and wind tunnel test, are required. Each of them applies thousands of strain tests on the aircraft surface and components. Thus, the existing methods make each aircraft go through the ground load calibration test, which has a heavy workload, a long cycle, and has the risk of accidental damage to the aircraft. Meanwhile, the method can only replace part of the strains. At least 50% of the strain still needs to be measured [26].

S2.3 Prediction method: from flight parameters to load

At present, there is no aircraft structural health monitoring method completely divorced from strain measurement. Realizing the reliable load prediction method from flight parameters entirely can get rid of the dilemma of strain gauges installation and maintenance and reduce the cost of aircraft structural health monitoring. Thus, in this work, we aim to design a load prediction method, which can calculate the aircraft load only by flight parameters.

Both the measurement-based method and the prediction-aided method are based on the prior knowledge that the load has relations to strains or flight parameters. Inspired by this, we explored the relationship between strains and flight parameters and came to a conclusion: There is Granger causality from flight parameters and strains! More than 70% pairs have the classical Granger causality and more than 80% pairs have the deep learning-based Granger causality. This finding indicates that strains can be predicted from flight parameters! Then, combining the equation between strains and load, we can use flight parameters to predict the load indirectly.

However, due to the huge amount of flight data and their highly nonlinear relations, the physical formulas between flight parameters and strains are hard to construct through expert knowledge. For such complex data relations, the data-driven approaches, especially deep learning methods (deep neural networks), are available [27]. Deep Learning models [28] have strong learning ability and flexible architecture. They can adaptively learn the implicit high-level feature representation of data, omit the steps of manually designing and extracting features, and then achieve high-precision prediction performance. At present, deep learning methods have achieved great success in many fields such as medicine and industry [29, 30]. But in the field of the aviation industry, there are few load prediction practices based on deep learning methods. Finnish researchers predicted the load of the rear fuselage wall panel, wing leading edge, rear beam, and vertical tail connection joint of F/A-18 aircraft [31]. But it only realized the prediction of partial strain, which still needs to be combined with the measured strains. And the method can only achieve the goal of less than 5% error in some special cases. The noisy data, the various relations, and the need for interpretation make the current deep learning methods hard to be directly applied for load prediction.

The flight data is special and needs the exclusive preprocessing method for the accuracy of the final prediction. The aviation industry is sophisticated and the load prediction is sensitive, where the error is required to be less than 5% [32]. However, small differences between flight parameters may cause large prediction differences [33]. Due to noise and electronic interference, flight parameters have data loss and distortion. The low data quality affects the prediction accuracy of the deep learning model.

One single deep learning model is limited by the diversity of relationships between flight data. Flight parameters reflect not only the load change but also the change of flight attitude. In the flight course, the aircraft will undergo many phases, such as takeoff, cruise, and landing. At each phase, the aircraft may also have many actions, such as turning and circling. In different flight attitudes, the same flight parameter could respond to different loads. That is, there are multiple data distributions and relations in one flight course. However, a single deep learning model is lack of ability to learn them all, as it is restricted by the premise of independent identically distributed (i.i.d) data, and learning of new knowledge will inevitably lead to the forgetting of old ones [34].

The interpretation of the method is important in the application of the aviation industry, but the deep learning model is not interpretable. The interpretability is important to model expansion, result analysis, and accuracy improvement. It can help aviation experts to verify and revise the method with physics knowledge. Besides, as structural health monitoring is directly related to flight safety and aircraft accidents, it requires more reliable and understandable methods [35]. However, most deep learning models are black box with complex data responses and feature representation. Nowadays, how to design an interpretable deep learning model is still an unsolved problem.

In this work, based on the potential nonlinear relationships among different flight data and the data similarity among aircraft in one fleet, we proposed a load prediction

method, which is a bold attempt to rely entirely on flight parameters to calculate the aircraft load after the model is established. We designed three key methods to solve the above difficulties: a complete flight data processing flow, a multi-model deep learning architecture and an important features based interpretation method. Besides, we implement a flight big data intelligent analysis software.

Different from the classical measurement-based method, our method predicts strains rather than measures strains after the model is built, which can avoid the error and cost caused by the strain measurement process; Different from the hybrid method, our method is an indirect process, where only one aircraft need to be created the strain-load equation, which can reduce the number of equations and avoid more complex tests for obtaining that equation. In a word, our load prediction method is data-driven: taking advantage of the existing data and learning the potential relationships among flight parameters, strains and load. In application, it can cut down some engineering processes by simultaneously reducing the use of unreliable strain gauges and avoiding potentially damaging tests for every aircrafts.

Supplementary References

[1] S Hochreiter and J Schmidhuber. Long short-term memory. *NEURAL COMPUT*, 9(8):1735–1780, 1997.

[2] Ninh Pham and Rasmus Pagh. A near-linear time approximation algorithm for angle-based outlier detection in high-dimensional data. In *ACM SIGKDD International Conference on Knowledge Discovery and Data Msining*, pages 877–885, 2012.

[3] Guolin Ke, Qi Meng, Thomas Finley, Taifeng Wang, Wei Chen, Weidong Ma, Qiwei Ye, and Tie-Yan Liu. Lightgbm: A highly efficient gradient boosting decision tree. In *Neural Information Processing Systems*, pages 3146–3154, 2017.

[4] Zeinab Falahiazar, Alireza Bagheri, and Midia Reshadi. Determining the parameters of DBSCAN automatically using the multi-objective genetic algorithm. *J INF SCI ENG*, 37(1):157–183, 2021.

[5] Scott M. Lundberg and Su-In Lee. A unified approach to interpreting model predictions. In *Conference on Neural Information Processing Systems*, pages 4765–4774, 2017.

[6] L. M. Nicolai and G. E. Carichner. Aircraft design. *American Institute of Aeronautics and Astronautics*, pages 435–465, 2010.

[7] G. Wild, L. Pollock, A. K. Abdelwahab, and J. Murray. The need for aerospace structural health monitoring: A review of aircraft fatigue accidents. *INT J PROGN HEALTH M*, 12(3), 2021.

[8] T. H. Skopinski, Wsj Aiken, and W. B. Huston. Calibration of strain-gage installations in aircraft structures for the measurement of flight loads. *Technical Report Archive & Image Library*, 1952.

[9] Norbert Fürstenau, Douglas D. Janzen, and Walter Schmidt. *Flight Tests of Fiber-Optic Interferometric Strain Gauges for Load Monitoring of Aircraft Structures*, pages 305–309. Springer US, Boston, MA, 1995.

[10] D. G. Marinaro, P. Mcmahon, and A. Wilson. Proton radiation effects on mems silicon strain gauges. *IEEE T NUCL SCI*, 55(3):1714–1718, 2008.

[11] Kai Han, Qian He, and Jia Zuo. Study on fault diagnosis for abnormal strain data in aircraft strength test. *Engineering & Test*, 56(04):7–12, 2016.

[12] Daniel B and Lothar S. Test of a fiber bragg grating sensor network for commercial aircraft structures. *Optical Fiber Sensors*, 16:55–58, 2002.

[13] Akira K and Yuji I. Optical fiber sensor based impact detection system for aircraft structures. In *29th ICAF Symposium, Nagoya*, 2017.

[14] Rutledge R S, Backman D S, and Lehman R A. Distributed sensing optical fibres for loads monitoring during full scale fatigue testing. In *29th ICAF Symposium, Nagoya*, 2017.

[15] Shienkman S and Kressel I. A condition based maintenance concept for monitoring helicopter composite rotor blades. In *30th ICAF Symposium, Nagoya*, 2019.

[16] Arjen K. Fibre optic sensing for structural health monitoring. In *30th ICAF Symposium, Krakow*, 2019.

[17] Tengfei Mu, Zhongjian Li, and Ximei Dai. Aircraft health monitoring technology. *Civil Aircraft Design and Resarch*, 03:35–41, 2020.

[18] X. Min, R. Bodik, and MD Hill. A flight data recorder for enabling full-system multiprocessor deterministic replay. *ACM SIGARCH Computer Architecture News*, 31(2):122–135, 2003.

[19] Molent L., Barter S., and Foster W. Verification of an individual aircraft fatigue monitoring system. *INT J FATIGUE*, 43:128–133, 2012.

[20] Tim F and Devinder M. F-35 joint strike fighter structural progno stics and health management: an overview. In *25th ICAF Symposium, Rotterdam*, 2009.

[21] Alexix F, Jean P K, and Alain S. The a400m usage monitoring function. In *28th ICAF Symposium, Helsinki*, pages 681–693, 2015.

[22] Kurdelski M, Reymer P, and Stefaniuk M. Service life extension program based on operational load monitoring system and durability test of the ageing fighter-bomber jet. In *29th ICAF Symposium, Nagoya*, 2017.

[23] Stephen D and Alex N. Verification of the raf c-130j structural health monitoring system through operational loads measurement. In *29th ICAF Symposium, Nagoya*, 2017.

[24] Hunt S R and Hebden I G. Validation of the eurofighter typhoon structural health and usage monitoring system. *European COST F3 Conference on System Indentification and Structural Monitoring*, pages 743–753, 2000.

[25] Haruhiro K and Tour F. Operational loads regression equation development for advanced fighter aircraft. *24th International Congress of the Aeronautical Sciences, ICAS*, 2004.

[26] Zhi Wang and Lei Wang. Review of life monitoring and structural health prediction management of foreign fighter jets. In *Aviation Safety and Equipment Maintenance Technology: proceedings of the Technical Symposium on Safety and Equipment Maintenance*, 2014.

[27] Shenda Hong, Yuxi Zhou, Meng Wu, Junyuan Shang, Qingyun Wang, Hongyan Li, and Junqing Xie. Combining deep neural networks and engineered features

for cardiac arrhythmia detection from ecg recordings. *China 2021 Top Cited Paper Award of IOP Publishing's (in Physiological Measurement)*, 40:054009 (12pp), 2019.

[28] LeCun Y, Bengio Y, and Hinton G. Deep learning. *Nature*, 521:436–444, 2015.

[29] Shenda Hong, Yuxi Zhou, Junyuan Shang, Cao Xiao, and Jimeng Sun. Opportunities and challenges of deep learning methods for electrocardiogram data: A systematic review. *Computers in Biology and Medicine*, 122:103801, 2020.

[30] Chenxi Sun, Shenda Hong, Moxian Song, Hongyan Li, and Zhenjie Wang. Predicting covid-19 disease progression and patient outcomes based on temporal deep learning. *BMC MED INFORM DECIS*, 21:45, 2020.

[31] Jarkko T and Tuomo S. Practical experience of neural network based fatigue life monitoring. In *28th ICAF Symposium, Helsinki*, pages 879–888, 2015.

[32] Yongjun Wang, Jiang Dong, and Hongna Dui. Aircraft structural load identification technology with high accuracy in sphm system. In *29th ICAF Symposium, Nagoya*, 2017.

[33] B. Y. Zhou, N. R. Gauger, J Hauth, H. Xun, and A. Guardone. Towards real-time in-flight ice detection systems via computational aeroacoustics and machine learning. In *AIAA Aviation 2019 Forum*, 2019.

[34] German Ignacio Parisi, Ronald Kemker, Jose L. Part, Christopher Kanan, and Stefan Wermter. Continual lifelong learning with neural networks: A review. *Neural Networks*, 113:54–71, 2019.

[35] Shenda Hong, Can Wang, and Zhaoji Fu. Gated temporal convolutional neural network and expert features for diagnosing and explaining physiological time series: A case study on heart rates. *COMPUT METH PROG BIO*, 200:105847, 2021.
